# T Cell Activation Machinery: Form and Function in Natural and Engineered Immune Receptors

**DOI:** 10.3390/ijms21197424

**Published:** 2020-10-08

**Authors:** Nicholas J. Chandler, Melissa J. Call, Matthew E. Call

**Affiliations:** 1 Structural Biology Division, Walter and Eliza Hall Institute, Parkville, VIC 3052, Australia; chandler.n@wehi.edu.au (N.J.C.); mjcall@wehi.edu.au (M.J.C.); 2 Department of Medical Biology, University of Melbourne, Parkville, VIC 3052, Australia

**Keywords:** T cell, immunoreceptor, CAR, structure, chimeric antigen receptor

## Abstract

The impressive success of chimeric antigen receptor (CAR)-T cell therapies in treating advanced B-cell malignancies has spurred a frenzy of activity aimed at developing CAR-T therapies for other cancers, particularly solid tumors, and optimizing engineered T cells for maximum clinical benefit in many different disease contexts. A rapidly growing body of design work is examining every modular component of traditional single-chain CARs as well as expanding out into many new and innovative engineered immunoreceptor designs that depart from this template. New approaches to immune cell and receptor engineering are being reported with rapidly increasing frequency, and many recent high-quality reviews (including one in this special issue) provide comprehensive coverage of the history and current state of the art in CAR-T and related cellular immunotherapies. In this review, we step back to examine our current understanding of the structure-function relationships in natural and engineered lymphocyte-activating receptors, with an eye towards evaluating how well the current-generation CAR designs recapitulate the most desirable features of their natural counterparts. We identify key areas that we believe are under-studied and therefore represent opportunities to further improve our grasp of form and function in natural and engineered receptors and to rationally design better therapeutics.

## 1. Introduction

**Figure 1:**
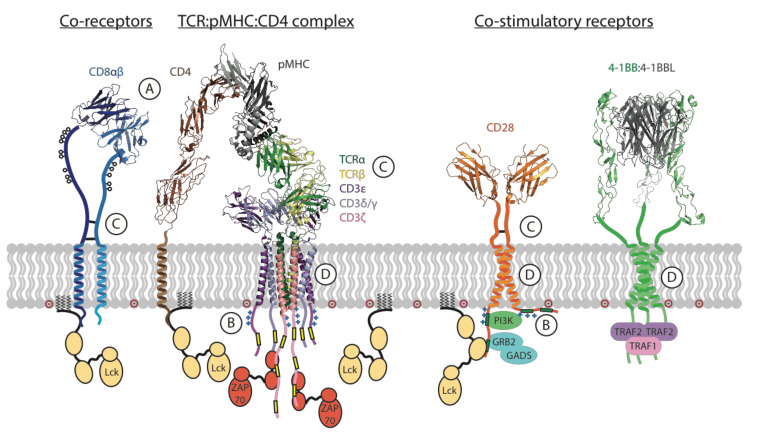
T cell activation following TCR recognition of stimulatory pMHC requires sensitivity enhancing co-receptor engagement of MHC (CD4 or CD8αβ) as well as co-stimulatory signals from constitutively expressed CD28 and several TCR induced co-stimulatory molecules (4-1BB depicted here). Yellow boxes represent ITAMs, green boxes represent non-ITAM stimulatory motifs. (**A**) Co-receptors CD4/CD8αβ engage MHC, dramatically increasing TCR sensitivity. (**B**) Positively charged tails interact with negatively charged lipid head groups. (**C**) Stalk cysteines facilitate interchain disulfide crosslinking. (**D**) Homo/hetero-typic TM interactions are vital to immunoreceptor assembly and function. Protein data bank (PDB) codes of structures shown in this figure: CD8αβ 2ATP, CD4/pMHC/TCRαβ 3TOE, TCR 6XJR (TCRαβ from 3TOE aligned against TCRαβ chains in 6XJR using pymol, 3TOE TCRαβ chains not shown), CD28 1YJD, 4-1BB/4-1BBL 6CPR.

**Figure 2:**
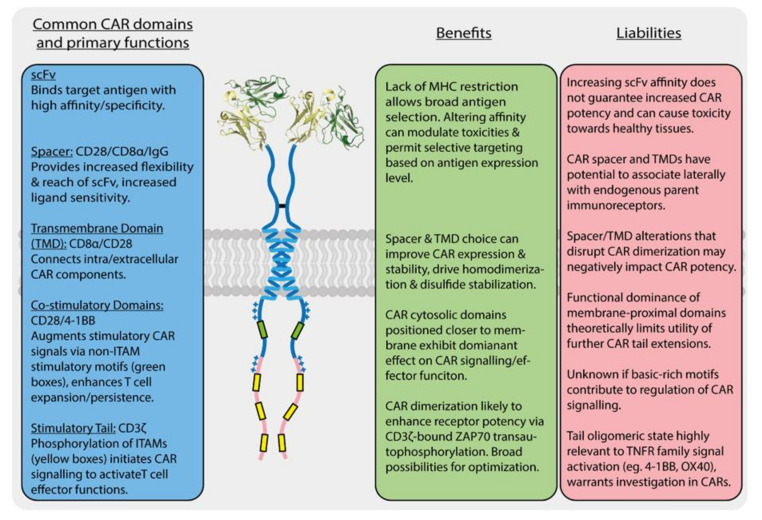
2nd Generation CAR constructs: the native receptor sequences commonly incorporated and the benefits and liabilities of those domains with regard to CAR function. Structure of the scFv domain is from PDB code 3H3B.

The key T cell functions, such as proliferation, target cell killing and cytokine secretion, are activated and regulated by a complex, multi-component molecular apparatus at the T cell surface. This activation machinery includes, at minimum, the eight-subunit T cell antigen receptor (TCR) [1,2], a co-receptor (CD4 or CD8) [3] and a costimulatory receptor (usually CD28) [4] (Figure 1). Various additional cell-surface molecules such as cytokine receptors and inhibitory receptors can positively or negatively influence the strength, quality and duration of activating signals. Given this level of complexity, it is remarkable that the basic outcomes of T cell activation can be effectively recapitulated for therapeutic benefit by engineered single-chain chimeric antigen receptors (CARs) [5,6]. A typical CAR couples an antibody-derived ligand-binding domain to spacer, transmembrane (TM) and signaling domains that are strung together using sequences from natural immune receptors (Figure 2). The development of this modular single-chain CAR format began at a time in the early 1990s before there was any detailed structural understanding of the molecules involved in T cell activation. The protein subunits making up the TCR complex had recently been identified [7,8], though neither their individual atomic structures nor their overall arrangement in the functional receptor were yet known, and the sequence of kinase-mediated events driving proximal signaling from the TCR was just being elucidated [9,10,11,12]. The molecular mechanisms of costimulatory signaling through CD28 were also just emerging [13]. Several groups had recently fused immunoglobulin and TCR genes to achieve antibody-like, major histocompatibility complex (MHC)-independent antigen recognition through the otherwise native, multi-subunit T cell signaling apparatus [14,15,16,17]. Much simpler single-chain chimeric receptor proteins had been used by others as research tools to show that the cytoplasmic tail of the TCR-associated ζ chain was sufficient to drive T cell activation [18,19,20]. The incorporation of single-chain antibody fragments (scFv) [21,22] to confer high-affinity tumor-antigen recognition and T cell activation through a single polypeptide chain by Esshar and colleagues [23] led to what we now regard as first-generation CARs, which were direct scFv-ζ fusions.

From the late 1990s, a rapidly growing collection of atomic structures of key signaling molecules and complexes was beginning to flesh out a more detailed understanding of natural immune receptor function. A great deal of this structural work focused on how the most common type of TCRs (αβTCRs) recognize their natural peptide: MHC ligands (reviewed in [24]), studies that have provided fundamental advances in understanding immune specificity but had arguably little impact on the parallel development of single-chain CARs. An enormous amount of structural and biochemical work has addressed the assembly and architecture of immune receptors, producing high-resolution structures of their key functional domains and yielding important mechanistic insights into how signaling platforms are nucleated and amplified at the inner face of the T cell plasma membrane. What lessons can be drawn from this body of work to better understand how current generation CARs function and how these functions can be improved through rational, structure-based design? Which features of natural immune receptor form and function are already well-embodied in current and emerging CAR designs? To what degree is it possible (or even desirable) to better recapitulate the more nuanced aspects of natural immune receptor signaling and regulation in engineered therapeutic receptors?

We begin to explore these questions by outlining the key features of natural immune receptors that relate to CAR design. For a more comprehensive treatment of the extensive literature relating to the structure and function of each receptor or protein family discussed below, we refer readers to recent reviews that are cited at appropriate places in the following sections and are also collected here [3,4,24,25,26,27,28,29,30,31] for convenience.

## 2. Key Features of Immune Receptors Involved in T Cell Activation

### 2.1. The TCR Is a Low-Affinity, High-Sensitivity Immune Receptor

The ability of T cells to effectively eliminate threats through recognition of foreign antigens relies on the remarkable functionality of the TCR (Figure 1). The TCR recognizes protein antigens only after they are processed and displayed as short peptides on the surface of an antigen-presenting cell (APC) or target cell by the proteins of the MHC [32,33,34,35], a phenomenon known as MHC restriction [36]. The TCR is incredibly sensitive: it can trigger calcium flux and cytokine release in response to a single stimulatory peptide-MHC (pMHC) ligand displayed alongside thousands of non-stimulatory self-peptides [37,38] and can activate target cell killing in response to as few as three stimulatory ligands [39]. The surprising observation that this high sensitivity and discriminating power comes despite relatively weak affinity of TCRs for pMHC complexes (with equilibrium dissociation constants K_D_ in the low μM range, approximately 1000-fold lower than a typical antibody–antigen interaction) was made as soon as the soluble proteins could be produced recombinantly [40,41] and has since been a topic of intense study. Higher-affinity TCR-pMHC interactions generally drive more potent T cell activation, but the relationship between affinity and potency is complex [29], with sometimes minimal differences between affinities for stimulatory and non-stimulatory antigens. Some of the difficulty in unravelling this relationship stems from the use of monomeric proteins in solution to measure the kinetics of interactions in three dimensions that, physiologically, occur only within the constraints of cell-to-cell contact interfaces in two dimensions with various additional forces and molecular interactions making contributions (see Section 2.9 below). Indeed, measurements of two-dimensional binding kinetics on intact T cells yield significantly higher affinities (due to slower off-rates) [42,43] and indicate that force [44,45] and even force directionality [46] play key roles in the unique binding characteristics governing TCR-pMHC interactions and their translation into activating signals. Although it is still unclear precisely how pMHC binding is transmitted across the T cell membrane [27], the complexity of the full TCR structure must be considered to begin to build an understanding.

### 2.2. The TCR Is a Complex of Eight Single-Spanning Membrane Proteins

The T cell receptor is an octameric assembly of type-I single-spanning membrane proteins arranged into four dimeric modules: the variable ligand-binding TCRαβ (in most T cells) module and the three invariant signaling modules CD3δε, CD3γε and ζζ. As described above, the TCRαβ module binds to pMHC ligands on APC or target cell surfaces, but these proteins lack intrinsic signaling capability and, as such, rely on the signaling modules to transmit information through their cytoplasmic immunoreceptor tyrosine-based activation motifs (ITAMs) [26,47]. The first soluble TCRαβ ectodomain structures were determined almost a quarter of a century ago [34,35], and the CD3 heterodimer [48,49,50,51] and ζζ homodimer [52] structures came in the following decade. However, their precise arrangement within the assembled complex was only recently captured in a cryogenic electron microscopy (cryo-EM) structure of the intact TCR octamer extracted from cell membranes in detergent [1] (Figure 1). This structure confirmed previous studies showing that a complex network of polar contacts within the eight subunit TM domains was a central determinant of assembly ([53,54,55] and references therein). Outside the membrane, the extracellular immunoglobulin (Ig) domains of CD3 heterodimers contact each other as well as the membrane-proximal Ig domains and connecting peptides of TCRαβ, confirming many of the predictions made from earlier mutagenesis studies (reviewed in [2,25,56]). The cytoplasmic tails were not resolved in the cryo-EM structure [1], consistent with what we know about their lack of stable structure and their dynamic interactions with lipids and signaling proteins (more on this in Section 2.6 below). This compact assembly involving a continuum of interactions throughout the extracellular and membrane-embedded domains is also consistent with proposals that structural rearrangements could mediate signal transduction through force-sensing [2,44,45,46,57,58], but a clear structural pathway from pMHC binding to initial phosphorylation events has yet to be identified [27].

### 2.3. Most Lymphocyte-Activating Receptors Have a Similar Multi-Subunit Architecture Based on TM Assembly

Like the TCR, the B cell antigen receptor (BCR) and activating receptors on natural killer (NK) cells and other innate lymphoid cells are assembled from a collection of ligand binding and signaling modules that associate via TM interactions [25]. While the CD3γε/CD3δε and CD79αβ signaling modules are unique to the TCR and BCR, respectively, other receptors integrate with a shared pool of structurally homologous signaling modules, including the TCR-associated ζζ dimer, the closely related antibody Fc receptor (FcR)γγ dimer and the NK cell modules DNAX-activation proteins 10 and 12 (DAP10 and DAP12). DAP12, in particular, serves as the signaling module for a large number of activating receptors on lymphoid as well as myeloid cells [59,60], and their association requires only a properly placed lysine residue in the receptor TM domain [61] to bind to a pair of aspartic acids in the DAP12 TM domain [62]. A similarly simple assembly unit is repeated three times within the TCR complex [1,53], twice within the natural killer group 2D (NKG2D)-DAP10 complex [63] and a single time in most other antigen-receptor systems [25]. This arrangement allows many different ligand-binding modules to couple with common signaling pathways, a feature that likely facilitated immune receptor diversification during evolution [59] and also represents an attractive platform for receptor engineering. However, not all of the sequences responsible for immune receptor TM associations are as well-defined, and it is likely that there are TM-mediated protein interactions we do not yet know about. These possibilities must be considered when repurposing segments containing TM domains for engineered receptors (more on this in Section 3.2 and Section 3.3 below).

### 2.4. The TCR Contains Signaling Motifs That Are Multiplied Both in Series and in Parallel

The remarkable sensitivity of the TCR is at least partly attributable to the large number of signaling motifs (ITAMs) that are associated with a single ligand-binding unit (TCRαβ) via the CD3δε, CD3γε and ζζ modules. The receptor complex contains 10 cytoplasmic ITAMs, each of which contains two phosphorylatable tyrosines within a consensus sequence **Y**xxL/Ix_6-8_**Y**xxL/I, where Y is tyrosine, x is any amino acid and L/I is leucine or isoleucine. The ITAM tyrosines are phosphorylated by the membrane-tethered lymphocyte-specific protein tyrosine kinase (Lck) (Figure 1) in a ligand-bound receptor, and this creates binding sites for the Src homology 2 (SH2) domains of the kinase zeta-associated protein-70 kDa (ZAP-70), a crucial promotor of TCR signaling, which serves to phosphorylate other ITAMs as well as multiple downstream signaling partners [26]. The ζ chain contains three ITAMs in series (one after the other in a single polypeptide), making the ζζ dimer the most potent signaling module with six ITAMs in total. The CD3γ, CD3δ and CD3ε tails each contain one ITAM, so the two CD3 heterodimers add four more ITAMs in parallel (at similar positions on laterally associated molecules) for a total of 10 ITAMs (Figure 1). For comparison, receptors that recognize far more abundant or polyvalent antigens generally possess either two or four signaling motifs [25]. It is notable that the ITAM-containing signaling modules in the TCR are all dimeric, a feature that may be linked to the requirement for phospho-ITAM-bound ZAP-70 to autoactivate through trans-phosphorylation [64,65], which is likely to happen most effectively when two ZAP-70 molecules are bound to ITAMs that are close together in a parallel configuration. This and other observations (see next section) indicate that the distinction between ITAMs that are multiplied in series versus in parallel is functionally relevant and may be a significant consideration for receptor engineering (see Section 3.4).

### 2.5. All ITAMs within the TCR Are Not Equal

The ITAMs in the different signaling chains of the TCR have different sequences, and there is a significant amount of empirical evidence that they are functionally non-equivalent in both their quantitative and qualitative contributions to TCR signaling. In a landmark study where mice with 25 different combinations of non-functional mutants versus normal ITAMs were examined for immune phenotypes, most combinations with less than seven functional ITAMs had T cell development defects that resulted in severe multi-organ autoimmunity [66]. However, the phenotype was only manifest in some combinations with six ITAMs and not with others, offering an indication that ITAMs were functionally non-equivalent. For example, mice with six ITAMs in parallel (WT CD3γδε but ζ with only the membrane-proximal ITAM intact) had normal peripheral T cell numbers but very low thymic cellularity and lethal autoimmunity, whereas mice with six ITAMs in series (WTζ but mutant CD3γδε, so actually two times three in series within the ζζ dimer) had reduced peripheral T cell numbers but closer-to-normal thymic makeup and no disease. Whether this is related to the specific ITAM sequences or to their configuration (or both) is not known. In another study examining the importance of ITAM diversity [67], no single ITAM sequence could recapitulate normal T cell development or function when used to replace all 10 instances in the TCR. Studies with synthetic peptides representing the phosphorylated forms of different ITAM sequences also clearly showed that they have different affinities for ZAP-70 and other key signaling molecules [26,68,69,70]. As discussed in the next section, some of the unique properties of different ITAMs may have to do with the context of other relevant motifs present in the different signaling tails and what types of molecular interactions these dictate.

### 2.6. Signaling Tails Participate in Complex Interactions with Lipids, Ions and Signaling Partners

The different signaling tails in the TCR and other immune receptors are not defined solely by the number or precise sequence of their ITAMs, and other motifs within the CD3 tails play significant roles in the regulation of signaling. Polybasic sequences (rich in lysine and arginine) within the CD3ε and ζ tails control binding to the negatively charged inner leaflet of the plasma membrane [71,72,73] (Figure 1B). This binding facilitates the sequestration of ITAM tyrosine residues into the lipid bilayer in their non-phosphorylated state [57], providing a layer of regulated accessibility to active Lck kinase that has been compared to the safety on the trigger of a gun [74]. Release of tail binding by, for example, force-induced structural changes that propagate through the TM domains of ligand-bound TCRs could thus provide a structural mechanism for initial receptor activation [2,27,57,58,75]. Signaling is amplified in part by the initial spike in intracellular free calcium (Ca^++^), which competes with anionic lipids for tail binding and further propagates ITAM release and phosphorylation [76]. CD3γ and CD3δ tails do not contain polybasic regions that bind lipids to sequester these ITAMs, but their phosphorylation appears to be indirectly regulated by the CD3ε tail transition [77]. The CD3ε tail may, in fact, be something of a master regulator of proximal TCR signaling: in addition to regulating ITAM phosphorylation, its dynamic membrane interactions control access to several other important regulatory motifs, the most well documented of which is a proline rich sequence (PRS) that recruits non-catalytic region of tyrosine kinase adaptor protein 1 (Nck) [78,79]. Very recent work also shows that the CD3ε tail can directly recruit both Lck [80] and its negative regulator C-terminal Src kinase (Csk) [81] through sequences that partially overlap with the full ITAM sequence, suggesting a much more complex role than previously appreciated.

### 2.7. T Cell Co-Receptors Enhance Sensitivity to Activating Ligands

The T cell co-receptors CD8 and CD4 bind to MHC molecules (class I and class II, respectively) through their extracellular domains and to Lck (the kinase that initiates TCR signaling) through their intracellular tails [3,82] (Figure 1A). When the TCR binds to a pMHC ligand on an APC or target cell, a co-receptor that is also bound to that MHC molecule will increase the local concentration of Lck and improve sensitivity by boosting the likelihood of ITAM phosphorylation. Lck is both myristoylated and palmitoylated, which localizes it to the inner leaflet of the lipid bilayer independently of co-receptors [83], but it also binds to CxC motifs in the CD4 and CD8 cytosolic domains [82], defining two pools of membrane-resident Lck based on whether it is “free” or co-receptor-associated. The textbook model is that recruitment of Lck through co-receptors is required to initiate TCR signaling, though this is not strictly true since artificial treatments such as CD3 cross-linking with antibodies that do not engage co-receptors are strong activators. In fact, recent time-resolved imaging experiments indicate that free Lck, as opposed to co-receptor bound Lck, is responsible for the earliest signaling events [84] in CD8^+^ T cells. This is consistent with a model in which a complex, biphasic interaction between TCR and CD8 is actually initiated by Lck-mediated recruitment of CD8 to the TCR [84,85] and not vice-versa.

While CD4 and CD8 perform similar functions, they differ significantly in their structure (see Figure 1) and affinity for MHC. The major form of CD8 on T cells is an αβ heterodimer (only CD8α binds Lck), with each chain contributing a single amino-terminal Ig domain on long stalk regions (~45 residues for CD8α, ~35 for CD8β) that connect to the TM domain. The stalks are rich in serine, threonine and proline and heavily in o-glycosylated and are therefore likely to be extended and at least somewhat rigid, and also contain cysteines that form intermolecular disulfide-bonds [3] (Figure 1A). While the Ig domains fold together to drive heterodimer formation, the TM domain of CD8α also plays a key role in assembly and trafficking and has a strong tendency to drive homodimer formation in the absence of CD8β [86]. CD4 is a monomer composed of four tandem Ig domains in the extracellular region. The structure of a full TCR-pMHC-CD4 ectodomain complex [87] (aligned with the cryo-EM structure of the full TCR complex [1] to generate the model in Figure 1) shows that the four Ig domains form an extended structure spanning a similar long dimension as the TCR-pMHC, with the distal (D1) domain bound to the membrane-proximal Ig domain of the MHC-II b chain. There is no analogous structure of a ternary TCR-pMHC-CD8 complex, but its similar MHC-binding mode [88] provides the basis for modeling the interaction. Human CD8αβ binds MHC-I with an affinity that is at least an order of magnitude lower than the affinity of an average TCR, yet it displays a cooperativity that stabilizes the TCR-MHC-I-CD8 complex in situ and significantly enhances pMHC discrimination [85]. Human CD4 binds MHC-II with even lower affinity, with a K_D_ estimated to be as low as ~2 mM [89]. Consequently, the stabilizing contribution of CD4 is likely to be negligible, yet participation of the CD4 co-receptor still affords significant additional sensitivity to activating ligands [37,90,91]. Interestingly, a glycine-based association motif in the TM domain of CD4 has been reported to contribute to this sensitizing function in a way that is Lck-independent [92], but the mechanism underlying this effect is unknown.

### 2.8. Co-Stimulatory and Inhibitory Receptors Control T Cell Priming and Suppression

In addition to TCR stimulation (signal 1) enhanced by co-receptor engagement, priming of naïve T cells is heavily dependent on signals delivered through CD28 (signal 2), a disulfide-linked homodimeric Ig superfamily (IgSF) protein and the prototypical co-stimulatory molecule [93,94] (Figure 1). CD28 is constitutively expressed on T cells, and its ligands CD80 (B7-1) and CD86 (B7-2) are expressed by activated APCs [4]. Ligand binding by CD28 induces phosphorylation of a (non-ITAM) tyrosine-based motif in its cytoplasmic tail by Lck, and this recruits phosphoinositide-3 kinase (PI3K) and adaptor proteins growth factor receptor-bound protein 2 (Grb2) and GRB2-related adaptor protein 2 (Gads). Additional proline-based motifs recruit other kinases including interleukin-2-inducible T cell kinase (Itk)/Tec kinase, Lck and protein kinase C (PKC)θ. Together, these proteins link to pathways that amplify TCR signaling and provide complementary signals to promote survival, support cytokine production and prepare T cells for the metabolic challenges of rapid proliferation. CD28 has also been reported to signal independently of TCR activation via a non-canonical nuclear factor kappa B (NF-κB) pathway that is particularly important for inflammatory cytokine and chemokine production [95]. This pathway is specific to the human CD28 tail sequence [96] and is not observed with mouse CD28. Activation through TCR and CD28 also induces expression of the structurally homologous cytotoxic T-lymphocyte-associated protein 4 (CTLA-4), another IgSF protein that forms a disulfide-linked homodimer, which competes for the same ligands as CD28 [97] and antagonizes its co-stimulatory activity [31]. This is just one of many regulatory mechanisms that act together to make T cell activation self-limiting, with other examples including additional induced inhibitory receptors (see below) as well as direct recruitment of negative regulators such as Lck inhibitor Csk [81], ZAP-70/extracellular-signal regulated kinase (ERK) inhibitor Rasal1 [98], PLCγ1 inhibitor Cish [99] and several E3 ubiquitin ligases [100] to the TCR and its proximal signaling platforms. The structural mechanisms governing CD28 signaling are complex: like CD3ε and ζ in the TCR, access to the CD28 tail is regulated by binding of a basic-rich sequence to the inner leaflet of the plasma membrane [101,102]. There is also evidence that TCR-mediated signals enhance CD28 affinity for its ligands [103,104] in an example of inside-out signaling reminiscent of integrin activation [105]. Structural changes involved in bi-directional signaling may be transmitted through the CD28 TM domain, which self-associates through a highly conserved polar motif Yx_4_T, where Y is tyrosine, x is any amino acid and T is threonine [106]. Notably, very similar Yx_4_T motifs also contribute to dimerization in CTLA-4 [106], ζ chain [52], FcRIγ [107] and TCRαβ [55]. Whether this motif serves a homologous functional purpose in each of these receptors remains to be determined.

In addition to CD28 and CTLA-4, there are several other co-stimulatory and inhibitory receptors that are expressed on T cells in response to TCR signaling [108]. The co-stimulatory receptor inducible T-cell costimulatory (ICOS) and the co-inhibitory receptors programmed cell death protein 1 (PD-1) and B- and T-lymphocyte attenuator (BTLA) are IgSF proteins that share similar domain architecture with CD28 but possess limited sequence identity and exhibit variability in their mechanism of action. Like CTLA-4, BTLA competes with activating receptors for ligand (Herpesvirus entry mediator (HVEM) in the case of BTLA) [109]. By comparison, PD-1 binds exclusively to the negative regulatory B7-family ligands PD-L1 and PD-L2 [110,111]. Both PD-1 and BTLA inhibit TCR signaling via direct interactions between cytosolic tail motifs and inhibitory SH2 domain-containing protein tyrosine phosphatases 1 and 2 (SHP-1 and SHP-2) [112,113]. In the case of ICOS co-stimulation, there appears to be considerable differences in mechanism of action compared to other members of the CD28 family. While the tail of ICOS is known to promote TCR signaling via a direct interaction with p85 and subsequent PI3K activation, a recent study showed that this co-stimulatory function was entirely dependent on Lck recruitment by the ICOS TM domain [114]. These findings indicate that the CD28 receptor family may not share a generalized mechanism of signaling and also highlight the potential for functionally relevant protein–protein interactions facilitated by domains other than the canonical signaling motifs encoded in cytoplasmic tails. A second group of co-stimulatory receptors induced following TCR signaling belong to the TNF receptor superfamily (TNFRSF) [28] and includes OX40, 4-1BB and HVEM. These receptors associate directly with the TRAF family of proteins upon ligand binding (Figure 1), promoting cell survival via the initiation of NF-κB signaling. TNFR ligands, ligand-bound TNFRSF receptors and TRAF proteins all adopt trimeric structures that are the base unit of activation [30]. Many more receptor–ligand interactions contribute to co-stimulatory or inhibitory signaling that impinges on T cell function at some stage of differentiation or in particular cellular contexts [4,28,108]. To date, CD28, 4-1BB, ICOS and OX40 represent the most common sources of co-stimulatory signaling motifs used in engineered receptors (see Section 3.5).

### 2.9. Immune Receptor Signaling in the Context of Cell–Cell Interactions

The receptor–ligand interactions described above all take place in the context of a highly orchestrated interface between a T cell and an APC that ultimately develops into a large structure known as the immunological synapse (IS) [115]. Initiation of activating signals occurs in smaller microclusters that form early after contact and contain kinase-associated TCR and CD28 (associated with ZAP-70 and PKCθ, respectively) [116], which coalesce to form the IS. The IS is a highly organized and dynamic structure containing TCR, co-receptors and co-stimulatory receptors surrounded by a ring of adhesion molecules such as lymphocyte function-associated antigen 1 (LFA-1) and CD2. The synaptic gap formed in the interior of the IS is small enough (~15 nm) to exclude large cell-surface proteins, most notably the inhibitory phosphatase CD45, whilst other receptor-ligand pairs with smaller dimensions, including TCR-pMHC and CD28-CD80/86, can diffuse and interact freely [117,118,119,120]. This “kinetic segregation” of a key phosphatase away from active signaling complexes allows kinases to dominate and supports the sustained signaling required for full T cell activation. The importance of the molecular dimensions across this cell–cell contact zone is highlighted by the significant loss of TCR sensitivity to ligand when either the TCR or MHC molecules are elongated through the experimental insertion of additional domains [118,121]. Concentrated within this crowded contact zone, receptors become laterally associated into larger platforms through assembly of signaling molecules and adaptors on their cytoplasmic tails, ultimately including negative regulators that modulate the strength and duration of signaling. The extent to which engineered receptors form this classical IS structure and/or are subject to the same regulatory mechanisms is an important area of study and has significant implications for CAR design (see Section 3.7 below).

## 3. Functional Consequences of CAR Design and Structure

As described in Section 1 above, the basic single-chain CAR design (scFv-spacer-TMD-tail) was established with little input from structural knowledge of immune receptors beyond the necessity of this modular, type-I membrane protein configuration. Since the demonstration that a first-generation (scFv-ζ) CAR could provide tumour-antigen-specific and MHC-independent T cell activation more than a quarter-century ago [23], the major design improvements that contributed to the ultimate therapeutic success of CAR-T cells have been the inclusion of a spacer domain for reach and flexibility [122,123] and the addition of costimulatory sequences in the cytoplasmic tails [124,125,126,127]. These features are discussed in more detail below. While the FDA approval of two such “second-generation” designs in late 2017 (tisagenlecleucel/Kymriah^®^ and axicabtagene ciloleucel/Yescarta^®^) for B cell malignancies has kept much of the effort to develop new CAR-T cell therapies focused on similar single-chain designs, many alterations have been investigated that can modulate their performance, and approaches are being developed that seek to improve safety and/or efficacy by incorporating lessons from the now-substantial body of knowledge around natural immune receptor structure, assembly, activation and regulation. The sections that follow are not intended to provide exhaustive coverage of the CAR design space (see detailed recent reviews [128,129,130,131,132,133,134]), and we will not focus heavily on the large number of completed or ongoing clinical trials (see detailed recent reviews [5,6,135,136]). Rather, we discuss what is known about single-chain CAR structure and function with reference to the above overview of natural immune receptors, identifying similarities and contrasts to highlight what we view as key benefits and liabilities of different features and domain configurations (summarized in Figure 2). We further touch on a few new receptor engineering approaches that incorporate this knowledge to expand the therapeutic applicability of next-generation CAR-T and related cellular immunotherapies for cancer.

### 3.1. CARs Are High-Affinity, Low-Sensitivity Immune Sensors

**Figure 3:**
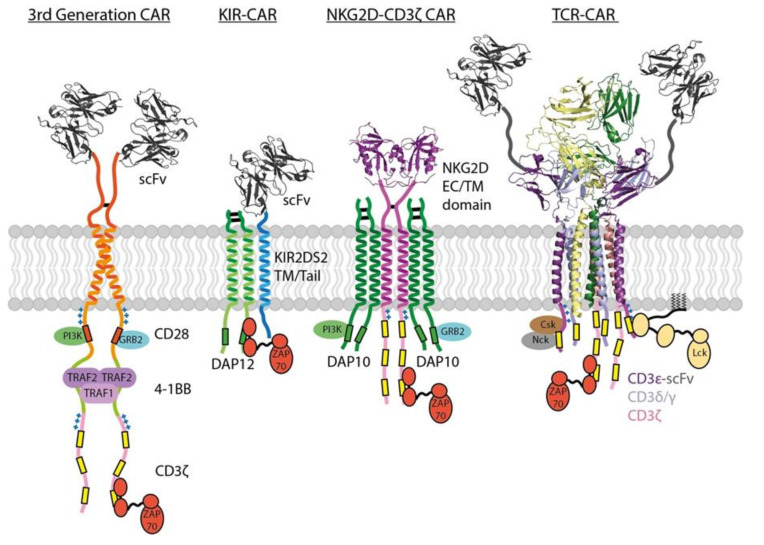
Novel design approaches to improving CAR function. 3rd Gen CARs use two or more co-stimulatory tails to improve signalling outcomes (OX40, ICOS, CD3ε also used). KIR-CARs and NKG2D CARs leverage native TM interactions to constitutively recruit endogenous signaling modules DAP12 and DAP10, respectively. TCR-CAR uses a CD3ε-scFv fusion to harness the high number of ITAMs and regulatory sequences within all six CD3 tails. PDB codes of structures shown are: scFv: 3H3B, NKG2D ECD: 1MPU, TCR: 6XJR.

CARs incorporate antibody scFv domains to bind tumor antigens without the MHC restriction imposed by TCRs and thereby enable recognition of native tumor antigens with, theoretically, any structure. As typical binding affinities for antibodies are in the low nM range, this also means that standard single-chain CARs bind antigens with approximately 1000-fold higher affinity than the mM TCR-pMHC interaction. Despite this fact, T cell activation through a CAR requires as much as 1000-fold higher antigen density than through a natural TCR recognizing pMHC [137]. Even in an experimental scenario where the CAR and TCR used the same antigen binding domain (an engineered TCR with high-nM affinity for a pMHC ligand) and with the CAR expressed at 10-fold higher surface levels than TCR, CAR-T cells were 10-fold to 100-fold less sensitive than T cells receiving signals through the high-affinity TCR complex [138]. This difference is undoubtedly related, at least in part, to the high ITAM content and structural complexity of the natural TCR and it underscores the fact that the parameters governing CAR signaling potency may be quite different from those uncovered through decades of studies on T cell activation. A recently reported TCR fusion construct approach [139] provides an alternative to standard CARs that leverages the signaling properties of the complete TCR complex by fusing the tumor antigen-binding scFv directly to the full extracellular domains of different TCR subunits, with CD3ε fusions proving most effective (see Figure 3). This approach yielded improved tumour killing compared to conventional CAR designs in vivo, however the comparative degree to which their engagement of endogenous TCR machinery influences T cell sensitivity and global gene expression remains to be determined.

Antibodies can have a very wide range of affinities for their antigens, and CAR-T cell sensitivity correlates directly with CAR scFv affinity for the target antigen [140,141,142]. This presents an opportunity for rational modulation of binding affinity with the aim of tailoring antigen sensitivity to a given disease setting. This approach has been used to reduce on-target, off-tumor toxicity in a human epidermal growth factor receptor 2 (HER2) positive tumor model where low antigen levels on healthy cells make them unintended targets for high-affinity CARs [142]. By generating a panel of HER2-specific scFvs with a range of reduced affinities, this study showed that intermediate-affinity CARs could selectively target HER2-high tumors while sparing healthy cells expressing lower levels of HER2. Modulating affinity of CARs to “tune” CAR-T cell function has also proven effective for other tumor antigens including epidermal growth factor receptor (EGFR), CD123 and CD38 in-mouse tumour models [143,144,145]. It is also clear from these studies that an upper limit exists above which increasing scFv affinity does not necessarily result in an increase in tumor-killing activity. This phenomenon was recently leveraged in a clinical trial investigating the effects of reduced-affinity CD19-specific CAR-T cells in the treatment of relapsed/refractory B-cell acute lymphoblastic leukemia (B-ALL) [146]. In line with the trial’s rationale, the reduction in affinity achieved comparably lower incidences of T cell potency-related side-effects such as cytokine release syndrome (CRS) [147] without sacrificing anti-tumor efficacy. Another very recent application closely related to this “affinity tuning” is “avidity tuning,” wherein low-affinity antigen-binding domains are used to make CARs whose activation thresholds are dependent on inducible receptor dimerization, multimeric antigens or the presence of two different antigens in very close proximity [148]. This approach is early in development, but it highlights the under-appreciated aspect of oligomeric state in CAR design.

### 3.2. Single-Chain Cars Are Not Monomeric at the Cell Surface

CARs are often depicted as monomers in cartoons, illustrating their domain organization, but nearly all current-generation single-chain designs actually form disulfide-linked homodimers. This is because the commonly used spacer and TM domains, included for the structural purposes of providing the reach/flexibility to bind antigens and a membrane anchor, respectively, come from immune receptor proteins that naturally dimerize through these sequences. First-generation CARs contained the entire ζ chain sequence [23], which forms a disulfide-stabilized dimeric interface through its TM domain [52,149]. Current FDA-approved anti-CD19 CAR-T therapies use either CD8α (tisagenlecleucel/Kymriah^®^) or CD28 (axicabtagene ciloleucel/Yescarta^®^) spacer and TM domains, both of which drive dimer formation in the native proteins and form stabilizing disulfide bonds in their stalk regions [86,106,150,151]. Other experimental CAR designs have used the short IgG antibody hinge (12 amino acids) or Fc region (one or two complete Ig domains) that drive disulfide-linked dimer formation in membrane-bound and secreted antibodies [141,152,153]. How much this dimeric format contributes to CAR function has not been widely investigated in design studies, but the observation that natural immune receptor signaling modules are dimeric [25] and the evidence that the key signaling kinase ZAP-70 requires a dimeric scaffold for optimal trans-autoactivation [64,65] suggests that this could be a crucial feature of engineered receptors. Some studies have shown that mutation of the stabilizing cysteines reduces CAR signaling potency [148,154], though it should be noted that disulfide bonds do not drive protein association, but only stabilize interactions driven by more extensive protein interfaces. These mutations are therefore unlikely to completely eliminate dimeric CAR interactions. A recent study comparing CD8α, CD28, CD4 and ζ spacer/TM regions in otherwise identical CAR constructs [151] found that CD8α, CD28 and ζ formed disulfide-linked dimers while CD4 did not, as expected from the structures of the source proteins. There was wide variability in surface expression, with a relationship of CD8α = CD28 > CD4 > ζ, and in vitro CAR-T cell activity tracked with expression level except that the CD28 spacer/TM consistently supported more potent activity than CD8α despite similar expression. This functional relationship has been documented by others [150,155,156], but since the spacer and TM domains are usually transferred together, it is not known whether these effects are traceable to one or the other (more on this in Section 3.3 below). Developing a clear rationale for the choice of these “purely structural” domains remains a challenge that will require a more complete understanding of what each of these sequences do in their native source proteins and thus what characteristics they confer in the context of CARs.

### 3.3. Repurposing Structural Domains from T Cell Proteins Has Both Benefits and Liabilities

Use of the entire ζ chain sequence in first generation CARs not only enabled homodimer formation, but also caused their incorporation into the TCR complex and formation of heterodimers with the endogenous ζ protein [154]. This is because the ζ TM domain contains sequences that direct both self-association and assembly with TCR [52,53,149]. A mutational analysis [154] based on the NMR structure of the ζζ TM homodimer [52] suggested that dimerization in a ζ-based CAR contributed more to sensitivity than its assembly with endogenous TCR components, but these functions of the ζ TM domain are difficult to separate [52,53,149] and both clearly made contributions to optimal CAR signaling. The ζ TM domain is no longer used in current-generation CARs, but some of the spacer/TM domains that are currently in use are much less well-defined in terms of their intrinsic molecular interactions and how these may contribute to differences in CAR functional properties. Both CD8α and CD28 stalk and TM domains contribute to dimer formation and stabilization, and biochemical studies suggest that the TM domains are independently capable of strong self-association in both cases [86,106]. Whether these can also drive lateral interactions with their endogenous counterparts is an open question, but if so, each could potentially contribute to CAR signaling in unintended ways, either through indirect recruitment of Lck (via recruitment of CD8α) or by amplification of costimulatory signals (via recruitment of endogenous CD28). The stalk domain of CD8α is also known to have variable patterns of serine/threonine o-glycosylation that are associated with different functional profiles during thymocyte development [157], presenting another pathway by which spacer/TM regions could contribute to variability in CAR function. Since CD8α and CD28 spacer/TM-containing CARs display similar levels of dimer formation and surface expression when compared in otherwise identical constructs [151], these types of mechanisms may explain their significant functional differences: the CD28 spacer/TM is consistently associated with higher antigen sensitivity, more cytokine production (corresponding with a more cytokine-related toxicity), more activation-induced cell death and higher levels of CAR-T cell exhaustion compared to the CD8α spacer/TM [150,155,156]. A striking example of the unpredictable nature of sequence optimization in these regions comes from a study examining small extensions of the CD8α-derived spacer/TM and intracellular membrane-flanking sequence in the prototypical CD19 CAR used in tisagenlecleucel/Kymriah^®^ [158]. Here, the authors identified a variant with an additional ten extracellular and four intracellular amino acids that exhibited reduced cytokine production while maintaining strong anti-tumor activity in a phase I clinical trial. The mechanism responsible for this reduced cytokine production is unknown, but this outcome emphasizes that some of the sequences being repurposed for CAR design could have functions we do not yet know about. For example, a recent study reported that the ICOS TM domain conferred more potent tumor suppression than the CD8α TM domain in otherwise identical CARs [159], an effect possibly related to the aforementioned ability of the ICOS TM domain to recruit Lck [114]. The molecular associations underlying these unexpected functional effects could be directly interrogated in an unbiased manner through the proteomic approach comparing CAR interactomes recently reported by Abate-Daga and colleagues [160]. However, this study was focused on comparing different costimulatory domain configurations rather than structural variations, and far larger systematic study will be required to analyze a comprehensive set of combinatorial possibilities (see Section 4 below).

Where the functions of spacer and TM domains are more completely understood, they can be used for rational design of receptor structures that potentially confer quantitatively and qualitatively distinct functional profiles compared with standard single-chain CARs (see Figure 3). One such approach involves the expression of a chimeric receptor composed of the entire NKG2D sequence fused to the ζ signaling tail [161]. In this configuration, the NKG2Dζζ fusion homodimer can assemble with two DAP10 dimeric signaling modules through well-defined TM interactions [63] to build a receptor complex that can potently activate T cells through six ζ ITAMs and four DAP10 costimulatory motifs. These were effective in mouse models of multiple myeloma and ovarian cancer expressing stress ligands recognized by NKG2D [162,163,164] but a phase I clinical trial showed limited NKG2Dζ CAR-T cell expansion and persistence [165], indicating that improvements are likely to be required for clinical viability. Others have investigated similar fusions using NKp44 and NKp30 [166], activating receptors that associate with DAP12 or ζ through their TM domains, respectively [25], to endow T cells with NK-like specificity. A related approach has been used to generate scFv-based CARs with the spacer and TM domains from activating NK cell receptor (killer cell immunoglobulin like receptor, two Ig domains and short cytoplasmic tail 2, KIR2DS2 )—KIR-CARs [167], which assemble with DAP12 through their TM domains [61,62,168] (Figure 3). KIR-CAR-T cells co-expressing DAP12 were very effective in a mesothelioma model and even outperformed standard second-generation single-chain CARs, despite having fewer ITAMs and no costimulatory domains [167]. This result highlights the possibility that building multi-chain receptor complexes that more closely resemble natural immune receptor structures could have tangible benefits that are not predictable based solely on the number of phosphorylation motifs engaged by target ligands.

### 3.4. CAR Potency Is a Function of ITAM Source, Number and Configuration

The first CAR designs used either ζ (three ITAMs) or FcRγ (one ITAM) sequences, and both were found to support basic receptor functions [23]. However, a later comparison showed that ζ-containing first-generation CARs were more potent inducers of T cell proliferation, cytokine production and cytotoxicity in vitro and in vivo compared to FcRγ-containing CARs [169], and nearly all second and third-generation CARs use ζ. This difference is presumably due to an increase in signaling potency with ITAM number, consistent with studies in the context of the natural TCR complex [64,66]. However, the notion that all ITAMs are not equal (see Section 2.5 above) is underscored in a recent study showing that even the three ITAMs within the ζ chain do not have equivalent activating potential in the context of a single-chain CAR [170]. An elegant set of experiments interrogating the functional consequences of ITAM number compared full-length ζ tail sequences possessing one, two or three functional ITAMs, where non-functional ITAMs had tyrosine to phenylalanine mutations. In an in vivo CD19^+^ tumor model, CARs with only a single ζ ITAM had enhanced anti-tumor efficacy compared to CARs with two or all three ITAMs intact. Single-ITAM containing CARs exhibited a clinically advantageous bias towards a memory T cell phenotype and increased persistence [170]. Interestingly, this effect was really only evident when the intact ITAM was the most membrane-proximal ITAM, and the authors showed that position, not sequence, was the distinguishing factor. This position may be the most potent because of its proximity to the membrane-associated Lck kinase, making it the only configuration that can support sufficient signaling when only one ITAM is available. These observations are generally in line with the idea that reduced signaling potency may increase CAR-T cell resistance to AICD and reduce exhaustion without compromising effector functions, particularly when tumor antigen expression is high. However, a more recent study of CD19^+^ tumors showed that this advantage is rapidly lost when tumor antigen expression is low [156]. At very low antigen levels (more than 100-fold lower than a representative CD19-high tumor), increasing the ITAM number to six via the linear concatenation of an additional ζ tail (this means 12 ITAMs in the dimeric CAR expressed at the cell surface) was required for measurable responses. These findings together indicate that antigen density sets a signaling potency threshold required for anti-tumor efficacy and emphasizes that both ITAM number and configuration can be empirically optimized for different disease settings to balance sensitivity with other features such as persistence. The potential utility of actively restraining CAR signaling activity is further underscored by a very recent study showing that reduced cytokine production and enhanced persistence were associated with addition of the CD3ε tail into a standard CD28-ζ CAR [81]. In its hemi-phosphorylated form (only the first tyrosine), the CD3ε ITAM recruits negative regulator Csk and attenuates signaling. An interesting side observation in this study was that the position of the CD3ε sequence within the CAR cytoplasmic tail also matters, since placement in the most membrane proximal position was effective but in the most membrane-distal position it abrogated CAR surface expression.

### 3.5. CAR Potency Is Also a Function of Co-Stimulatory Motif Source, Number and Configuration

First-generation CARs can induce robust target cell killing and significant cytokine production, but the in-vivo expansion and persistence required for anti-tumor efficacy in patients were not achieved until costimulatory sequences were added to yield the second-generation CARs that represent the current state-of-the-art in clinical application [171,172,173]. Preclinical studies investigating different sources and configurations of costimulatory sequences have rarely compared CAR constructs that were otherwise identical, largely because spacer and/or TM domains were often (but not always) transferred along with the costimulatory tails as these were varied [124,125,126,174]. Even when the co-stimulatory sequence used is constant, two studies comparing CD28-ζ and ζ-CD28 configurations, both of which concluded that CD28-ζ was superior, were each comparing a construct containing the CD28 TM and cytoplasmic domains with ones that used the CD8α [124] or ζ [125] TM domain. As described in Section 3.3 above, these sequences have their own unique and intrinsic benefits and liabilities, and this is a significant confounding factor when not accounted for systematically. This caveat notwithstanding, a comparison of CD19 CARs containing CD28, DAP10, 4-1BB or OX40 costimulatory sequences in a single study [126] found that the CD28 tail (inserted with its TM domain) uniquely supported antigen-specific in vitro T cell expansion and strong cytokine production. In the context of a mesothelin-targeted CAR, CD28 and 4-1BB tails both supported effective tumor control in mice, but CD28 (again with its TM domain) yielded faster eradication and 4-1BB (with CD8α TM domain) uniquely supported long-term CAR-T cell persistence [175].

These results have generally been borne out in the context of the FDA-approved CD19 CARs, for which there is now a great deal of patient data showing that while overall response rates are similar, 4-1BB-containing CARs mediate slower tumor rejection but cause less cytokine toxicity and persist for longer [6]. Only very recently have the molecular mechanisms underpinning these functional differences begun to be systematically dissected. By comparing signaling downstream of CD28 and 4-1BB tail sequences in the context of otherwise identical CD19 CARs, one study [176] found that enhanced Lck recruitment amplifies both basal and antigen-induced phosphorylation in the CD28-containing CAR, while the unique ability to recruit phosphatase SHP1 (in a THEMIS-SHP1 complex) suppresses basal phosphorylation and attenuates antigen-induced phosphorylation in the 4-1BB-containing CAR. Importantly, the authors went on to show that engineering SHP1 recruitment into the CD28-containing CAR could ameliorate cytokine toxicity without sacrificing overall efficacy, emphasizing the utility of gaining detailed mechanistic understanding for engineering better therapies.

The incorporation of two or more costimulatory domains has recently given rise to a third generation of CAR designs [174,175,177,178] (Figure 3). While there is evidence that these can combine, for example, the best features of CD28 (rapid expansion and killing) and 4-1BB co-stimulation (lower cytokine production and longer persistence) into a single-chain CAR, how much clinical advantage they will have in different disease settings remains to be seen. There are interesting indications that the configuration of multiple costimulatory sequences is crucial. In a study comparing 4-1BB-ICOS-ζ with ICOS-4-1BB-ζ configurations (both using CD8α spacer/TM), the membrane-proximal sequence appeared to dominate the resulting CAR-T functional profile [159]. This is in line with similar observations in the context of ITAM position (see Section 3.4 above) and may be an effect of proximity to the membrane-associated Lck kinase. However, an ICOS-4-1BB-ζ configuration that also used the ICOS TM domain was superior to both of these in vivo, possibly due to the independent ability of this TM domain to directly recruit Lck discussed in Section 3.3 [114]. Another study examining different CD28 + 4-1BB configurations showed that the most effective combination used a CD28-ζ CAR with the 4-1BB ligand co-expressed to engage endogenous 4-1BB in cis (on the same cell) [174], which was consistently better than a single-chain CAR incorporating both co-stimulatory sequences in series and suggests that parallel engagement is advantageous. A very recent study investigating anti-HIV CARs also showed that T cells co-expressing two standard CD28-ζ and 4-1BB-ζ CARs (with the same antigen-binding domain) outperformed those expressing a single third-generation CAR containing both co-stimulatory domains [179], again suggesting that parallel engagement may be superior and that each costimulatory sequence may need to be in the most membrane-proximal position to exert maximum influence on signaling.

### 3.6. Additional Modifications to CAR Signalling Tails

While a great deal of research is dedicated to optimizing ITAM and co-stimulatory motif source, number and configuration in CAR cytoplasmic tails, the field has also begun to explore other modifications that can provide additional benefits. For example, second- and third-generation CARs engage both activating (signal 1) and co-stimulatory (signal 2) pathways but still rely on autocrine and paracrine action of cytokines (signal 3) to provide additional signals supporting proliferation and effector/memory cell differentiation [180,181]. A recent study incorporated sequences derived from the IL-2Rβ chain into a second-generation CD19-CD28-ζ CAR, including the Box 1/2 motifs that recruit JAK1 kinase and the YLSL motif that recruits STAT5 as well as an extra TRHQ motif in the ζ tail sequence to recruit STAT3 [182]. CAR-T cells made using this construct displayed enhanced expansion, persistence and tumor control in mice. Another very recent study [183] reports a novel modification replacing all lysines in first- and second-generation CD19 CAR tails with arginines (KR mutations) to eliminate ubiquitin-mediated down-regulation while maintaining the charge balance in the cytoplasmic sequence that impacts on regulatory interactions with the plasma membrane (see Section 2.6 above). These authors report that CD19-41BB-ζ CAR^KR^-T cells maintained higher CAR surface levels and exhibited enhanced expansion, persistence, tumor control and central memory T cell differentiation in vivo. These are but two examples of novel and creative modifications, based on clear functional rationales, that are representative of the types of design improvements that may be expected in the next generation of CAR proteins.

### 3.7. CAR Signalling in the Context of Cell–Cell Interactions

As in conventional TCR-mediated T cell activation, CAR–antigen interactions take place in the confined space between T cells and target cells with closely apposed membranes and a large number of adhesion molecules and other cell-surface receptors and regulatory proteins. While CAR engagement drives formation of concentrated areas of receptors and proximal kinases at the target cell contact point in a similar way to conventional T cells, the structure and behavior of the CAR-T cell IS is quite different. One study using mouse CD8^+^ T cells co-expressing the OT-I TCR and a second-generation HER2-CD28-ζ CAR showed that the CAR IS was smaller, less organized and shorter-lived that the TCR IS, but resulted in faster recruitment of lytic granules and therefore faster killing [184]. What features of CAR signaling are responsible for this effect is not yet known, though affinity for antigen and length/structure of the spacer domain are clear structural candidates that could affect molecular segregation. Quantitative measures of synapse quality such as degree of CAR clustering, actin polymerization and lytic granule concentration may be useful predictors of in vivo CAR-T cell performance [185,186] and thus a greater understanding of the factors at play could be important for developing efficient pipelines for prioritizing new candidate designs. A recent study using a third-generation CD19-CD28-41BB-ζ CAR showed that CAR-T cell synapses were unstable in a large fraction of cells, but those that effectively concentrated receptor-ligand microclusters into a central region were able to exclude the key phosphatase CD45 and build effective signaling platforms [187]. This study began to dissect the roles of different adaptor molecules known to be important in conventional T cell IS formation, and further work in this area promises a more complete picture of CAR IS structure and dynamics and its role in CAR-T cell function.

## 4. Concluding Remarks

Systematic CAR optimization will require exploration of a very large combinatorial space that becomes rapidly cumbersome when preclinical animal studies are involved and impractical at the stage of phase I clinical trials. While the modularity of the single-chain CAR format makes it readily amenable to optimization of each domain, the rapid accumulation of potentially beneficial modifications poses a significant challenge to the development of a broadly applicable theoretical framework to guide engineered receptor design. This point is well illustrated by reviewing the number of different approaches that have reported promising progress towards two of the most significant challenges in CAR-T cell therapy [5,6]: reducing toxicity and increasing CAR-T cell persistence. The sections above, while not comprehensive, cite examples of affinity and avidity tuning via the antigen-binding domain, exchange or modification of spacer and TM domains (with ill-defined effects on both homo- and hetero-typic molecular interactions), changes to the source, number and/or configuration of intracellular signaling domains, both activating (ITAMs) and co-stimulatory, and incorporation of completely new types of modifications that bring in additional signaling pathways or enhance CAR protein lifetime, all of which have shown significant promise for improving CAR-T cell safety or efficacy. There are widely variable levels of rationale for these modifications (some have arisen from purely empirical explorations and some had very clear theoretical underpinnings), and comparisons among them are confounded by alterations to the functions of multiple domains at once, whether intentional or unintentional. Furthermore, the degree to which these modifications can be combined for additive or synergistic benefit has rarely been investigated, and even comparison of their benefits and liabilities is complicated by fundamental differences among studies in design and delivery of the CAR gene vectors, cellular composition of the product used for preclinical and clinical studies, combination with additional genetic modifications or pharmacological agents and a host of other variables. There is ample evidence to suggest that CAR-T cell therapies will need to be optimized separately for many different types of cancer, and just the design of the receptor proteins themselves, separate from additional cell modifications such as targeted disruption of inhibitory genes, inclusion of safety mechanisms, etc., represents a vast variable matrix. As we develop a deeper understanding of the structural and functional characteristics of each CAR protein component and how they work together, this large parameter space will begin to contract into a set of design rules that can be used to predictably modulate CAR function to suit the specific demands of different disease contexts.

## Abbreviations

AICD	Activation-induced Cell death
APC	Antigen-presenting cell
B-ALL	B-cell Acute Lymphoblastic Leukemia
BCR	B-cell receptor
BTLA	B- and T-lymphocyte attenuator
CAR	Chimeric antigen receptor
CRS	Cytokine release syndrome
Csk	C-terminal Src kinase
CTLA-4	Cytotoxic T-lymphocyte-associated protein 4
Cryo-EM	Cryogenic electron microscopy
DAP EC	DNAX-activating protein Extracellular
ERK EGFR GADS	Extracellular signal-regulated kinase Epidermal growth factor receptor GRB2-related adaptor protein 2
Grb2	Growth factor receptor-bound protein 2
HVEM	Herpes virus entry mediator
ICOS	Inducible T-cell costimulatory
IgSF	Immunoglobulin superfamily
IS	Immune Synapse
ITAM	Immunoreceptor tyrosine-based activation motif
Itk	Interleukin-2-inducible T-cell kinase
JAK	Janus kinase
KIR	Killer Ig-like receptor
KIR2DS2 Lck	Killer Ig-like receptor two Ig domains and short cytoplasmic tail 2 Lymphocyte-specific protein tyrosine kinase
MHC	Major histocompatibility complex
NK NKG2D	Natural killer Natural killer group 2 member D
pMHC	Peptide-bound major histocompatibility complex
PDB LFA-1	Protein Data Bank Lymphocyte function-associated antigen 1
NMR	Nuclear Magnetic Resonance
Nck	Non-catalytic region of tyrosine kinase adaptor protein 1
PD-1 PI3K	Programmed death receptor 1 Phosphoinositide 3-kinase
PKC	Protein kinase C
scFv	Single-chain variable fragment
SH2 SHP-1	Src-homology 2 Src homology 2 domain-containing protein tyrosine phosphatase 1
STAT	Signal Transducer and Activator of Transcription protein
TCR	T cell receptor
THEMIS TM	Thymocyte-expressed-molecule Transmembrane
TNF	Tumor necrosis factor
TNFR TNFRSF TRAF	Tumor necrosis factor receptor Tumor necrosis factor receptor Superfamily TNF receptor associated factor
ZAP-70	Zeta-associated protein 70
